# Lung Cell Toxicity of Metal-Containing Nanoparticles

**DOI:** 10.3390/nano12173044

**Published:** 2022-09-02

**Authors:** Hanna L. Karlsson, Andrea Hartwig

**Affiliations:** 1 Institute of Environmental Medicine, Karolinska Institutet, 171 77 Stockholm, Sweden; 2 Department of Food Chemistry and Toxicology, Institute of Applied Biosciences (IAB), Karlsruhe Institute of Technology (KIT), 76131 Karlsruhe, Germany

Among the various nanomaterials present in society, many contain metals or metal compounds. Highest exposure levels are present at workplaces, with inhalation being the main exposure route. This Special Issue on “Lung Cell Toxicity of Metal-containing Nanoparticles”, in the MDPI journal *Nanomaterials*, features seven original papers. They cover a wide range of metal-containing nanoparticles (NPs) and use traditional in vitro models, as well as non-traditional models, including a continuous flow system to understand dissolution, long-term in vitro exposure, co-cultures, and the use of air–liquid interface exposure. Two studies also use in vivo models. The studies highlight different mechanisms of underlying toxicity, including the role of cell uptake, dissolution in model fluids and within the cell, particle size and surface functionalization, and furthermore explore changes in gene expression and epigenetic alteration.

One of the nanoparticle types previously shown to cause high toxicity is copper oxide (CuO) NPs. These particles were studied in both an in vitro model by Strauch et al. [1] and in an in vivo model by Rossner et al. [2]. In the study by Strauch et al. [1] the impact of endocytosis and lysosomal acidification for the toxicity of CuO particles in both nano- and micrometer size was explored using BEAS-2B cells. The results showed, for example, that both sizes of particles caused changes in gene expression of several genes (especially *HMOX1*, *HSPA1A*, *MT1X*, *SCL30A1*, *IL8,* and *GADD45A*), with CuO NPs showing far more pronounced alterations. These changes were completely abolished when the uptake was inhibited. Interestingly, inhibition of lysosomal acidification clearly reduced transcriptional changes induced by the NPs, whereas the effect caused by those in micron-size were not affected. Rossner et al. [2] also focused on changes in gene expression following exposure to CuO NPs, but in this case a mice inhalation model was used (3 days, 2 weeks, 6 weeks, and 3 months) followed by whole genome transcriptome profiling using next generation sequencing. A significant transcriptomic response was observed already after 3 days of exposure; here, genes related to collagen formation were affected whereas longer treatments caused changes related to the immune response. The expression of miRNA was also explored, and no changes were noted after 3 days of inhalation whereas some modifications were observed at prolonged exposures.

Another study focusing on genome wide gene expression changes is the study by Gliga et al. [3]. Here, the authors investigated changes after six weeks of exposure of BEAS-2B cells to nickel (Ni) and nickel oxide (NiO) NPs in comparison to soluble NiCl_2_. Despite the limited cellular uptake, exposure to NiCl_2_ resulted in the largest number of differentially expressed genes. Several top enriched pathways for NiCl_2_ were defined by upregulation of interleukin-1A and -1B, as well as Vascular Endothelial Growth Factor A (*VEGFA*). Gene expression changes noted for all three Ni exposures included genes coding for calcium-binding proteins (*S100A14* and *S100A2*), as well as *TIMP3*, *CCND2*, *EPCAM*, *IL4R,* and *DDIT4*. The results also showed that all Ni exposures caused DNA strand breaks whereas no induction of micronuclei was noted.

The study by Vales et al. [4] also investigated genotoxicity in terms of DNA strand breaks and micronuclei formation after exposure of BEAS-2B but in this case with a focus on gold (Au) NPs in different sizes (5 and 20 nm) and surface functionalization. The cytotoxicity of ammonium-functionalized Au NPs limited the doses to be applied. The genotoxicity results showed that the 20 nm ammonium Au NPs were most efficient in terms of causing DNA strand breaks whereas the PEGylated particles induced more micronuclei. The authors concluded that neither functionalization nor size could clearly explain the genotoxic effects of the Au NPs.

Except from the in vitro studies using BEAS-2B cells already mentioned, the study by Cappellini et al. [5] used another lung model, i.e., a co-culture of A549 cells and differentiated THP-1 cells. The aim of the study was to investigate the cytotoxic and inflammatory potential of CeO_2_ NPs (NM-212) and to compare the impact upon submerged and air–liquid interface exposure, respectively. The cellular dose was carefully examined in both models using inductively coupled plasma mass spectrometry (ICP-MS). Limited inflammatory effects were observed in the doses tested in the co-culture but a clear effect of IL-1β release was noted in monocultures of macrophage-like THP-1. 

Wiemann et al. [6] also used macrophages (NR8383 alveolar macrophages) to compare the toxicity of kaolin and bentonite (nanoclay NM-600) in similar size to explore the possibility to group these aluminosilicates. A rat model was also used, and quartz (DQ12) was included as a reference material. The results showed that bentonite was far more toxic in vitro, as well as in the rat lungs where the early effects clearly exceeded those of kaolin (as well as of quartz). The authors thus concluded that the results argue against a common grouping of aluminosilicates.

In addition to these studies using various cell and animal models, the study by Keller et al. [7] instead used an acellular flow system to explore the pulmonary lysosomal dissolution of nanomaterials. First the authors explored the most optimal ratio of particle surface area per volume flow-rate of three very different materials: ZnO (quick dissolution), TiO_2_ (very slow dissolution), and BaSO_4_ (partial dissolution). Next, they used the optimized model to also study non-nanoforms of the same substances and to test aluminosilicates. The results showed, for example, that in case of TiO_2_ and BaSO_4_, the nanoform and the non-nanoform exerted the same dissolution rate when normalized to the specific surface area. Furthermore, in case of aluminosilicates, two different nanoforms of Kaolins had the same dissolution rate, while ion release was faster from Bentonite. For all aluminosilicates, a faster leaching of Si than of Al was observed.

This collection of articles emphasize the usefulness in combining different models and techniques, as well as competences for the increased understanding of lung cell toxicity of metal-containing nanoparticles.
